# Nebulized Bacteriophage in a Patient With Refractory *Mycobacterium abscessus* Lung Disease

**DOI:** 10.1093/ofid/ofac194

**Published:** 2022-04-12

**Authors:** Rebekah M Dedrick, Krista G Freeman, Jan A Nguyen, Asli Bahadirli-Talbott, Mitchell E Cardin, Madison Cristinziano, Bailey E Smith, Soowan Jeong, Elisa H Ignatius, Cheng Ting Lin, Keira A Cohen, Graham F Hatfull

**Affiliations:** Biological Sciences, University of Pittsburgh, Pittsburgh, Pennsylvania, USA; Biological Sciences, University of Pittsburgh, Pittsburgh, Pennsylvania, USA; Division of Pulmonary and Critical Care Medicine, Johns Hopkins University School of Medicine, Baltimore, Maryland, USA; Division of Pulmonary and Critical Care Medicine, Johns Hopkins University School of Medicine, Baltimore, Maryland, USA; Division of Pulmonary and Critical Care Medicine, Johns Hopkins University School of Medicine, Baltimore, Maryland, USA; Biological Sciences, University of Pittsburgh, Pittsburgh, Pennsylvania, USA; Biological Sciences, University of Pittsburgh, Pittsburgh, Pennsylvania, USA; Division of Pulmonary and Critical Care Medicine, Johns Hopkins University School of Medicine, Baltimore, Maryland, USA; Division of Clinical Pharmacology, Johns Hopkins University School of Medicine, Baltimore, Maryland, USA; Division of Infectious Diseases, Johns Hopkins University School of Medicine, Baltimore, Maryland, USA; Department of Radiology, Johns Hopkins University School of Medicine, Baltimore, Maryland, USA; Division of Pulmonary and Critical Care Medicine, Johns Hopkins University School of Medicine, Baltimore, Maryland, USA; Biological Sciences, University of Pittsburgh, Pittsburgh, Pennsylvania, USA

**Keywords:** *Mycobacterium abscessus*, bacteriophage, mycobacteriophage, phage therapy

## Abstract

An elderly man with refractory *Mycobacterium abscessus* lung disease previously developed anti-phage neutralizing antibodies while receiving intravenous phage therapy. Subsequent phage nebulization resulted in transient weight gain, decreased C-reactive protein, and reduced *Mycobacterium* burden. Weak sputum neutralization may have limited the outcomes, but phage resistance was not a contributing factor.

*Mycobacterium abscessus* is an emerging opportunistic pathogen of increasing clinical significance [1]. The medical management of *M abscessus* lung disease is often complicated due to limited numbers of effective antibiotics, prolonged duration of therapy, and frequent treatment-related adverse events [2]. Bacteriophage therapy, in which viruses that infect bacteria are utilized as targeted antimicrobial agents, is a therapeutic strategy that has been applied to treat challenging infections [3–5]. Poor treatment outcomes in *M abscessus* infections have prompted exploration of mycobacteriophage therapy for these organisms, although the optimal patient selection, dosing, and route of phage administration for nontuberculous mycobacteria (NTM) remain unclear [5, 6].

Two recent case reports [7, 8] described divergent outcomes with the use of an intravenous (IV) mycobacteriophage cocktail against *M abscessus*. The first patient was a 15-year-old girl with cystic fibrosis (CF) whose post–lung transplant course was complicated by treatment-refractory disseminated *M abscessus* infection, which was successfully controlled with the use of an IV mycobacteriophage cocktail [7]. A second patient, an immunocompetent elderly man with non-CF bronchiectasis and refractory *M abscessus* subspecies *massiliense* lung disease [8], was treated with the identical phage cocktail, dose, and route (IV) as the first patient [7]; however, he developed a robust phage neutralizing antibody-mediated response that temporally associated with *M abscessus* treatment failure and led to its discontinuation. Here we report the outcomes of this patient after switching the route of phage administration to aerosolized delivery.

An 81-year-old man with non-CF bronchiectasis and refractory macrolide-resistant *M abscessus* lung disease was treated for 6 months with an IV cocktail of 3 mycobacteriophages (Muddy, BPsΔ*33*HTH_HRM10, and ZoeJΔ*45*) in addition to a multidrug antibiotic regimen, as previously described [8]. An IV route of phage administration initially was selected due to concerns that anatomic abnormalities from lung cavitation could limit phage access to infected lung tissues. IV phage therapy resulted in a transient decline in *M abscessus* burden in sputum at month 1 (Figure 1*C*), with subsequent rebound in mycobacterial colony-forming units (CFUs) that temporally correlated with the emergence of a robust anti-phage immunoglobulin M and immunoglobulin G (IgG) neutralizing antibody response, specific to the phages used therapeutically [8]. No alternative phages that infect this strain were available that might have circumvented the immune response [9]. After a continuous 6 months of twice-daily IV treatment, phage therapy was discontinued due to lack of sustained clinical effectiveness.

**Figure 1.**
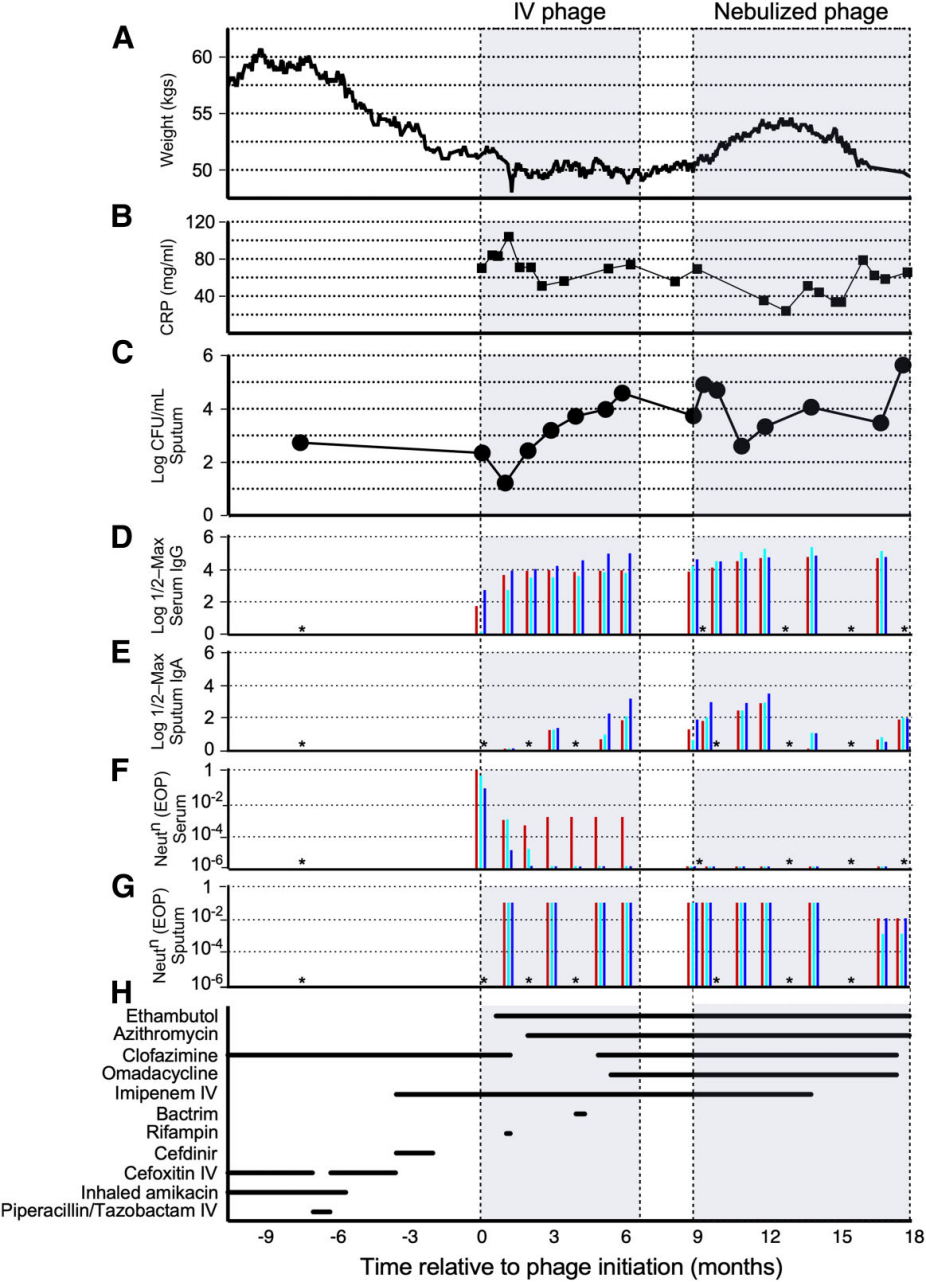
Clinical response to intravenous and nebulized mycobacteriophage therapy for refractory *Mycobacterium abscessus* lung disease. A, Patient weight. B, Serum C-reactive protein level. C, Mycobacterial burden as measured in *M abscessus* log colony-forming units (CFUs)/ mL^−1^ in sputum. D and E, Serum immunoglobulin G (IgG) (D) and sputum immunoglobulin A (panel E) log half-maximal antibody levels against phages Muddy, BPsΔ*33*HTH_HRM10, and ZoeJΔ*45* (blue, aqua, and red, respectively) determined by enzyme-linked immunosorbent assay. F and G, Serum (F) and sputum (G) phage neutralization after 24 hours of incubation with phage in vitro expressed as efficiency of plating (EOP) relative to a control with no addition. An EOP of 1 reflects no neutralization; reduction in EOP reflects phage neutralization. H, Timeline of companion antibiotic exposure. Details provided in Supplementary Materials. Sputum CFU^−1^ counts, serum IgG, and serum neutralization during intravenous administration are as reported previously [8]. *Samples not available or not tested. Abbreviations: CFU, colony-forming units; CRP, C-reactive protein; EOP, efficiency of plating; IgA, immunoglobulin A; IgG, immunoglobulin G; IV, intravenous; Neut^n^, neautralization.

To overcome serum neutralization and to increase phage delivery to the site of infection, the patient was started on nebulized administration of the same triple phage cocktail. Regulatory and institutional approval was obtained from the United States Food and Drug Administration and the Johns Hopkins Institutional Review Board and written informed consent was obtained prior to initiation. While continuing multidrug antibiotic therapy for NTM lung disease (Figure 1*H*), the phage cocktail was administered twice daily via nebulization using a Philips Respironics InnoSpire Essence Compressor Nebulizer System at a dose of 1 × 10^9^ plaque-forming units in 3 mL of 0.9% normal saline. Safety and efficacy monitoring during nebulized therapy included serial clinical and microbiological assessments, in-home spirometry with the Microlife PF 100 digital peak flow and forced expiratory volume in 1 second (FEV_1_) meter, and serial chest computed tomography (CT). Standing weight was recorded regularly by the patient (Figure 1*A*) and quantitative assessment of *M abscessus* counts in expectorated sputum was calculated approximately monthly as the mean CFUs/mL^−1^ (Figure 1*C*). Serial *M abscessus* isolates were assessed for changes in antibiotic susceptibility and for acquisition of phage resistance.

Nebulized phage delivery was relatively safe and well tolerated. Prior to initiation of nebulized phage, baseline lung function was measured with an FEV_1_ of 1.71 L (Supplementary Figure 1*A*). Following initiation of nebulized phage therapy, FEV_1_ transiently increased to 2.13 L on day 3, and later remained relatively stable over time, with a 0.12 L decline during the 9-month nebulized treatment period (Supplementary Figure 1*B*). Changes in companion antibiotics during phage therapy were motivated by adverse events suspected to be antibiotic-related. These events included development of eosinophilia, prompting discontinuation of IV imipenem, and nausea and mild liver function test abnormalities, leading to discontinuation of omadacycline and clofazimine (Figure 1*H* and Supplementary Figure 2). There were no other clinically significant differences in blood counts or serum chemistries (Supplementary Figure 2*A*). Parenchymal lung abnormalities on chest CT appeared relatively unchanged over time, with only minor interval changes (Supplementary Figure 3). A brief (5-day) interruption in the use of nebulized phage therapy occurred in month 7 of nebulized phage due to development of self-limited small volume hemoptysis, a not uncommon event in patients with bronchiectasis. Hemoptysis was not temporally related to phage administration, resolved spontaneously without intervention, and did not recur after resuming nebulized phage.

With initiation of nebulized phage therapy, there was a reduction in subjective sputum production. By approximately 3.5 months into nebulized phage therapy, the patient’s body weight increased by 8.7% for a total weight gain of 4.3 kg (9.5 lbs) from a prenebulized phage baseline weight of 49.7 kg (109.5 lb) (Figure 1*A*). C-reactive protein (CRP) decreased from 69.3 mg/L prenebulized phage to a nadir of 23.9 mg/L 4 months into nebulized phage (Figure 1*B*). While *M abscessus* burden in sputum fluctuated (Figure 1*C*), weight gain during nebulized phage therapy correlated temporally with a relative quantitative reduction in *M abscessus* in sputum and a decrease in CRP—consistent with an improved systemic response to infection with the use of nebulized phage.

Approximately 4 months after initiation of nebulized phage delivery, these apparent benefits from phage administration dissipated, and the subsequent 7 weeks of treatment were accompanied by weight loss, increase in CRP, and rising sputum bacterial counts (Figure 1*A*–1*C*). Because of concerns for potential physical destruction and phage loss with the jet compression-type nebulizer [10, 11], administration was switched after 7.5 months to a vibrating mesh-type nebulizer (Philips Respironics InnoSpire Go), although this change did not improve clinical outcomes (Figure 1).

To determine if treatment failure was caused by the emergence of phage resistance, *M abscessus* isolates recovered 7 and 8 months after the start of phage nebulization were tested for phage susceptibility; these specimens had similar profiles to earlier isolates and remained fully sensitive to all 3 phages in the regimen (Supplementary Figure 4*A*). The emergence of bacterial resistance to phage therapy was not observed, nor were there changes in antibiotic susceptibilities (Supplementary Table 1). Sputum samples were tested for an immunoglobulin A (IgA)–mediated response to the phages (Figure 1*E*, Supplementary Figure 4*C*), and a weak but notable response to all 3 phages prior to nebulization was observed, which fluctuated after the start of nebulization but did not increase consistently or substantially; sputum IgA titers remained about 100-fold lower than serum IgG levels to the same phages (Figure 1*D*). This sputum IgA reactivity may have occurred, in part, as a response to the 9 months of prior IV phage administration. However, sputum samples showed only weak phage neutralization (Figure 1*G*, Supplementary Figure 4*B*), which was about 4 logs lower than observed with serum samples 8 months post–phage nebulization (Figure 1*F*).

The clinical progression following the switch to aerosolized phage administration mirrors what was observed after the start of IV administration: some benefits initially, followed by subsequent dissipation of those clinical gains. For IV administration, the emergence of strong neutralizing antibody response to the phages was temporally correlated with increases in mycobacterial burden and rises in CRP (Figure 1*B* and 1*C*). After the start of aerosolized delivery, weight gain, fall in CRP, and decline in mycobacterial load were initially encouraging. These clinical improvements lasted longer (3–4 months) than those observed after start of IV therapy (1–2 months). However, these gains with nebulized phage were again only transient, and the mechanisms underlying the limited duration of the benefits of phage aerosolization in this patient are unclear. We anticipated that emergence of phage resistance might occur, but after 8 months of nebulization—and 15 months of total phage therapy—the strain remained fully sensitive to phages Muddy and BPsΔ33HTH_HRM10, and with only a slight reduction in sensitivity to ZoeJΔ*45* (Supplementary Figure 4*A*), which was also observed after IV treatment alone [8]. The failure to encounter phage resistance in *M abscessus* clinical isolates is consistent with in vitro studies [9] and is an encouraging indicator of the clinical utility of phage therapy against *Mycobacterium* infections. Aerosolized treatment failure did not result from antibody-mediated phage neutralization either, as only relatively weak sputum IgA responses to the phages were observed, and only mild neutralization (Figure 1*E* and 1*G*). However, neutralization did increase at later times (7 and 8 months) after the start of nebulization, and it is plausible that this contributed to limitation of treatment effect.

This case study highlights the challenges with using aerosolized bacteriophages for controlling *Mycobacterium* infections. Some patients may see clinical improvement and long-term benefits [7] whereas others may see little or only transitory health gains. It is likely that pathological details such as the specific sites of infection within the lung, access of the phages to infected foci, and host responses are all important factors that warrant further investigation.

## Supplementary Data

Supplementary materials are available at *Open Forum Infectious Diseases* online. Consisting of data provided by the authors to benefit the reader, the posted materials are not copyedited and are the sole responsibility of the authors, so questions or comments should be addressed to the corresponding author.

## Supplementary Material

ofac194_Supplementary_Data
