# Supplementary material for: Nebulized Bacteriophage in a Patient With Refractory Mycobacterium abscessus Lung Disease
Source: Open Forum Infect Dis. 2022 Apr 12;9(7):ofac194. doi: 10.1093/ofid/ofac194 (PMC9251665; doi:10.1093/ofid/ofac194)
Supplement: ofac194_Supplementary_Data [file ofac194_supplementary_data.zip › Supplemental Figures.pdf]

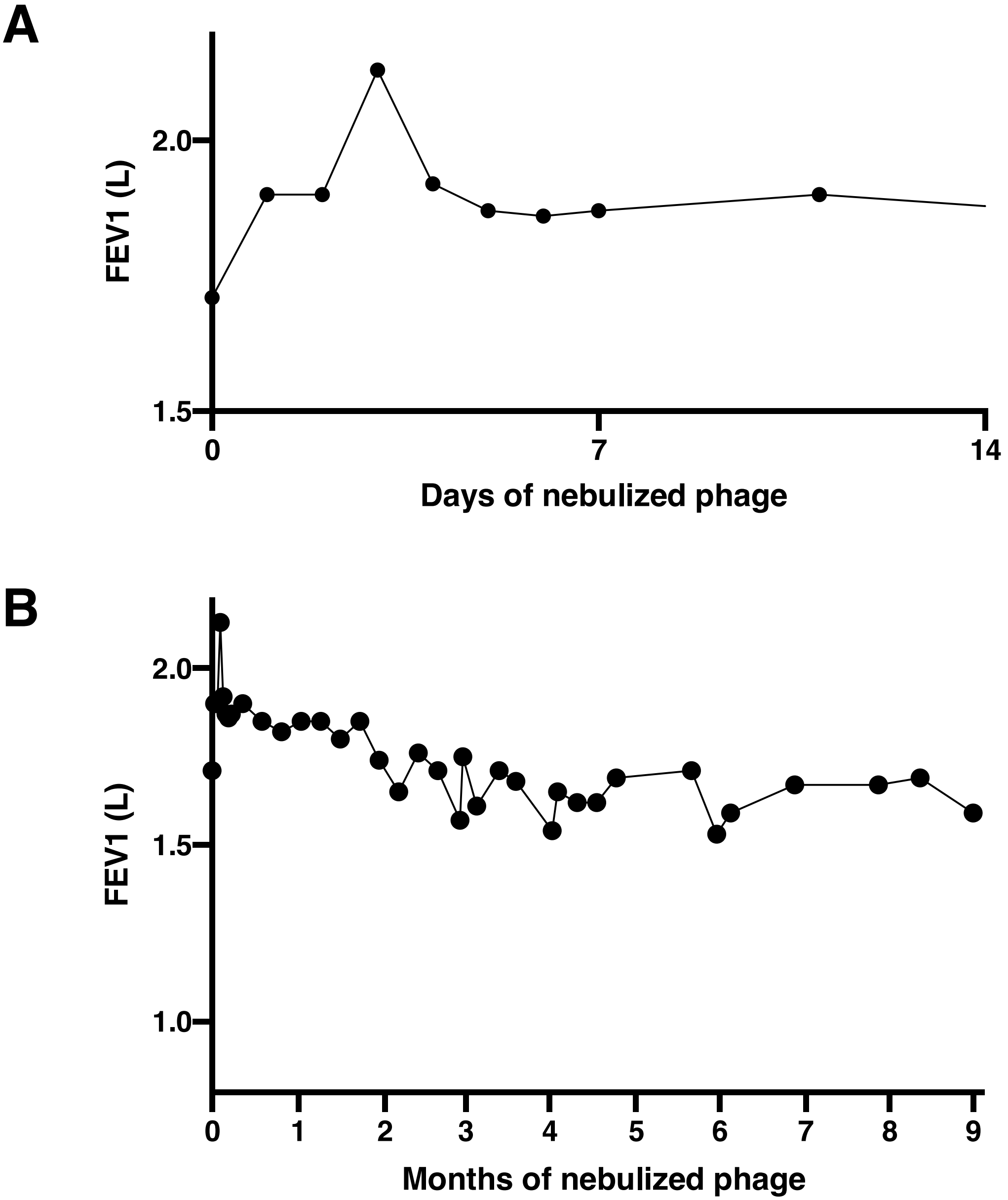

Figure S1. FEV1 during nebulized phage treatment. Lung function as FEV1 in liters during **A**) the first 14 days of nebulized phage therapy, and **B**) 9 months of nebulized phage therapy.

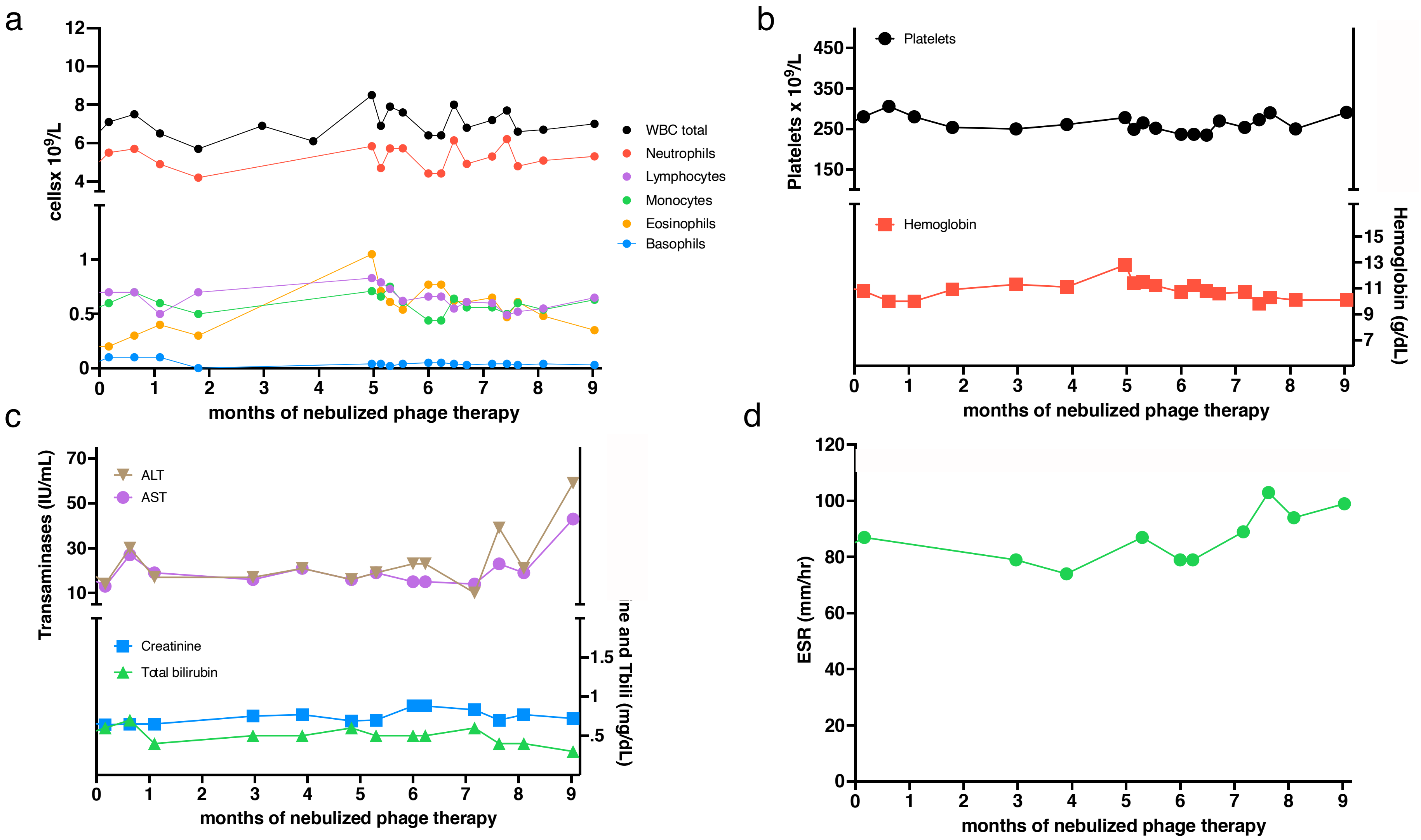

Figure S2. Clinical indicators during nebulized phage treatment. Safety assessments during phage treatment included regular monitoring of complete blood counts with differential, comprehensive metabolic panels, and inflammatory markers. During nine-months of nebulized phage treatment there were no clinically significant differences attributable to the use of nebulized phage in **A)** white blood cell (WBC) total counts and differentials, **B)** hemoglobin and platelets, **C)** liver function (alanine transaminase, ALT; aspartate transaminase, AST; and total bilirubin) or kidney function (creatinine), and **D)** erythrocyte sedimentation rate (ESR).

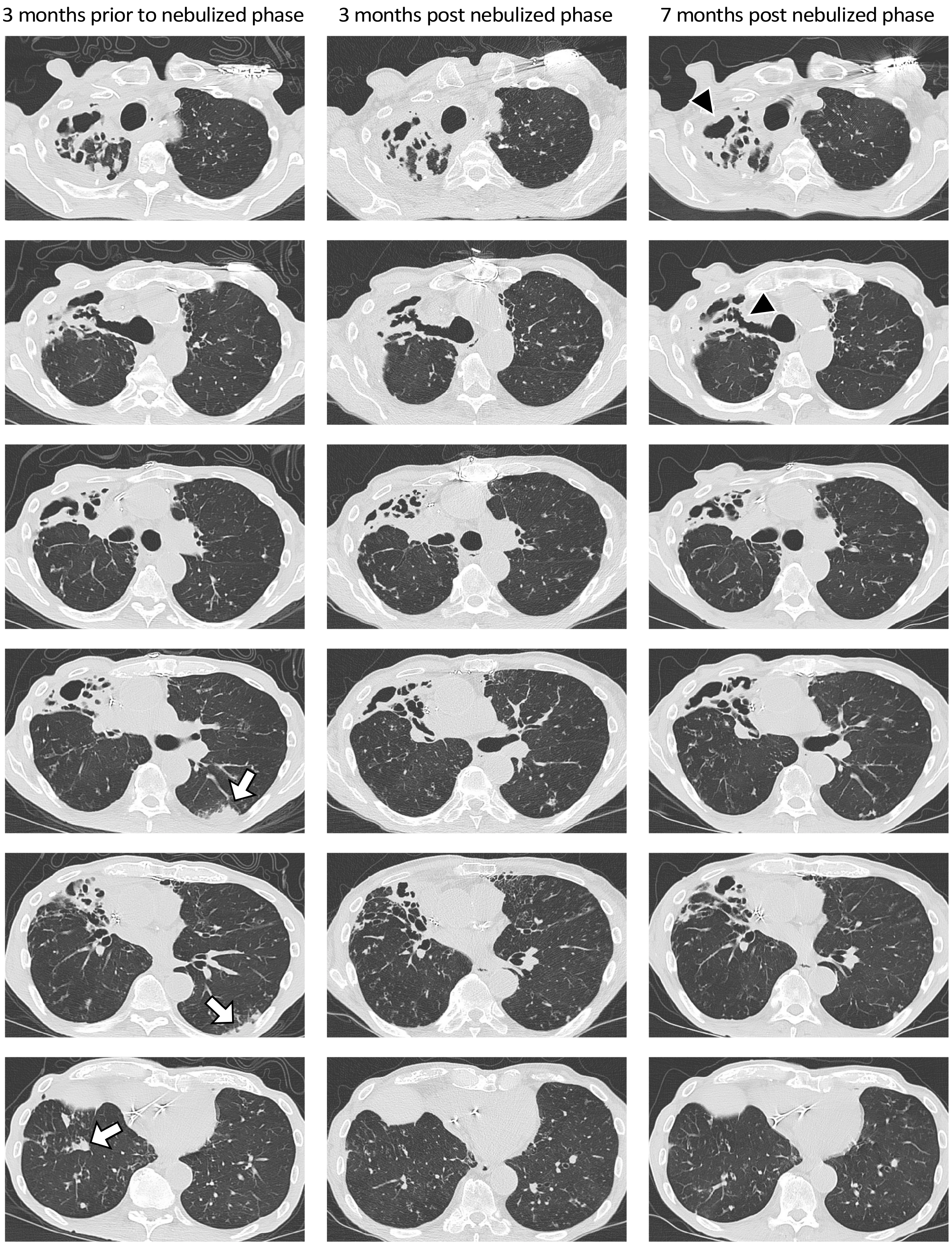

Figure S3. Chest CT images during nebulized phage therapy. Non-contrast high resolution chest CT scans were performed three months prior to nebulized phage treatment and repeated after nebulized phage treatment initiation at three- and seven-months. Chronic airway disease and parenchymal fibrosis most severe at the right upper lobe (black arrowheads) were largely stable throughout nebulized phage therapy, whereas the acute patchy consolidations in the bilateral lower lobes resolved (white arrows).

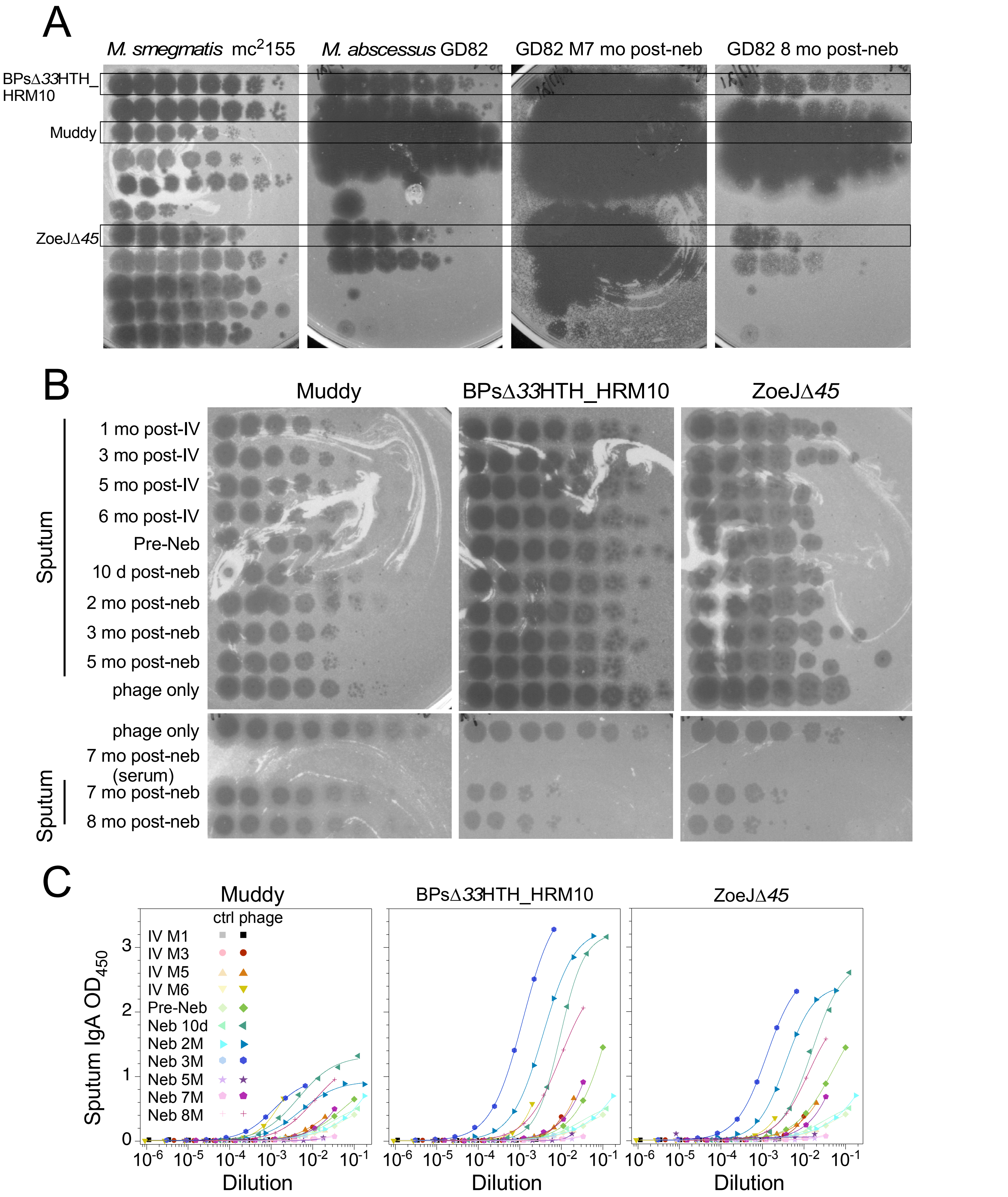

Figure S4. Phage susceptibilities, phage neutralization, and anti-phage ELISAs. A. Phage lysates were 10-fold serially diluted and spotted onto lawns of *M. smegmatis* mc^{2}155, *M. abscessus* GD82 (original patient isolate) and GD82 recovered either 7 or 8-months after the start of nebulization. The three phages used therapeutically are boxed. **B.** Either sputum or serum samples (as indicated) were incubated with phages Muddy, BPsΔ33HTH_HRM10 or ZoeJΔ*45*, incubated for 24 hours, then 10-fold serially diluted and plated onto lawns of *M. smegmatis* mc^{2}155. **C.** ELISA curves using sputum and a secondary antibody specific for IgA binding.
