# Supplementary material for: Nebulized Bacteriophage in a Patient With Refractory Mycobacterium abscessus Lung Disease
Source: Open Forum Infect Dis. 2022 Apr 12;9(7):ofac194. doi: 10.1093/ofid/ofac194 (PMC9251665; doi:10.1093/ofid/ofac194)
Supplement: ofac194_Supplementary_Data [file ofac194_supplementary_data.zip › Supplementary Materials 3-24-22.pdf]

Supplemental Materials

Methods

Bacterial strains and phage susceptibility screening.

M. smegmatis mc^2155 and *M. abscessus* GD82 isolates were grown and tested for phage susceptibility as previously described [8].

Mycobacteriophage cocktail preparation and administration.

Phages were grown, harvested, purified, and dialyzed as previously described [8]. Undetectable levels of endotoxin were observed in any dialyzed sample using the EndoZyme II (Hyglos GmbH) assay. Accugen, Inc confirmed that each sample was sterile by performing USP-71 assays. The three phages were combined to form a cocktail, with each phage at $1x10^{11}$ PFU ml^{-1}. The cocktail was diluted with 0.9% normal saline for a dose of $1x10^9$ PFUs in 3 ml and this dose was administered by nebulization twice daily.

Phage neutralization assays and ELISAs.

Serum and sputum samples were tested for phage neutralization, as well as IgA and IgG responses using methods reported previously [8].

Mycobacterial counts in sputum and MIC determination of GD82 isolates.

Expectorated sputum was processed, and MIC determination was completed as reported [8].
