# Supplementary material for: Nebulized Bacteriophage in a Patient With Refractory Mycobacterium abscessus Lung Disease
Source: Open Forum Infect Dis. 2022 Apr 12;9(7):ofac194. doi: 10.1093/ofid/ofac194 (PMC9251665; doi:10.1093/ofid/ofac194)
Supplement: ofac194_Supplementary_Data [file ofac194_supplementary_data.zip › Table S1_Mab_MIC_phage_011422.pdf]

Table S1. Antibiotic susceptibility of *M. abscessus* isolates

	Time relative to nebulized phage treatment initiation							
**Antibiotic[1]**	**Day 0**	**Day 10**	**Month 1**	**Month 2**	**Month 3**	**Month 5**	**Month 7.5**	**Month 8.5**
Trimethoprim/sulfamethoxazole	>8/152	>8/152	>8/152	>8/152	>8/152	>8/152	>8/152	>8/152
Ciprofloxacin	>4	>4	>4	>4	>4	>4	>4	>4
Moxifloxacin	>8	>8	>8	>8	>8	>8	>8	>8
Cefoxitin	>128	64	64	64	64	64	128	128
Amikacin	>64	32	32	32	32	16	32	64
Doxycycline	>16	>16	>16	>16	>16	>16	>16	>16
Tigecycline	>4	4	>4	4	>4	4	>4	>4
Linezolid	>32	>32	>32	>32	>32	32	>32	>32
Imipenem	>64	64	64	64	64	64	>64	>64
Cefepime	>32	>32	>32	>32	>32	32	>32	>32
Amoxicillin/clavulanate	>64/32	>64/32	>64/32	>64/32	>64/32	>64/32	>64/32	>64/32
Ceftriaxone	>64	>64	>64	>64	>64	>64	>64	>64
Minocycline	>8	>8	>8	>8	>8	>8	>8	>8
Tobramycin	>16	>16	>16	>16	>16	>16	>16	>16
Clarithromycin (3 days)	>16	>16	>16	>16	>16	>16	>16	>16
Clarithromycin (14 days)	>16	>16	>16	>16	>16	>16	>16	>16

[1]Antibiotic minimum inhibitory concentration (MIC) determinations (in µg/mL) against *M. abscessus* GD82 were determined pre-nebulized phage therapy, and monthly during nebulized phage therapy using a standard antibiotic panel for rapidly growing mycobacteria (Sensititre RAPMYCO™).
